# Supplementary material for: Are carbon emissions trading and green financial instruments synergistic? -Comprehensive quantitative research based on content analysis
Source: PLoS One. 2024 Mar 7;19(3):e0298601. doi: 10.1371/journal.pone.0298601 (PMC10919601; doi:10.1371/journal.pone.0298601)
Supplement: S1 Data — (ZIP) [file pone.0298601.s001.zip › Data/地级市Z_F工作报告.zip!/░▓╗╒/│╪╓▌╩╨/└┤╫╘╣1⁄2╓┌║┼í╛┬φ┐╦╩2╛▌═°í┐_2017.docx]

各位代表：

现在，我代表市人民政府，向大会报告工作，请予审议，并请市政协委员和其他列席人员提出意见。

一、2016年工作回顾

过去的一年，全市人民在市委的坚强领导下，全面贯彻党的十八大和十八届三中、四中、五中、六中全会精神，深入贯彻习近平总书记系列重要讲话特别是视察安徽重要讲话精神，统筹做好稳增长、促改革、调结构、惠民生、防风险各项工作，经济社会发展总体平稳、稳中有进，较好地完成了市三届人大五次会议确定的各项目标任务，实现了“十三五”良好开局。预计，全市生产总值583亿元、增长8.2%，财政收入突破100亿元、增长4.5%，固定资产投资652亿元、增长8.6%，社会消费品零售总额221亿元、增长11.5%，城乡居民人均可支配收入分别增长8%、9%。

一是扎实推进供给侧结构性改革，经济稳定增长根基不断夯实。制定实施推进供给侧结构性改革、促进经济健康发展等政策措施，建立重点工作、重点项目月调度制度，实行问题、措施、责任“三项清单”管理，320个省亿元以上重点项目完成投资286亿元。总投资百亿元的长九（神山）灰岩矿综合开发项目以及双威电子、卡尔菲特产业园等20个亿元以上工业项目开工建设，世界村生命源水一期、普洛康裕制药二期和三期、超威新能源产业园、科成电子等32个亿元以上工业项目竣工投产，九华山直升机低空游览、醉山野原生态文化休闲度假区、富贵陵阳九华大峡谷等3个亿元以上文化旅游项目建成开放，贵航特钢、冠华黄金入列2016安徽省制造业百强，全年净增规模工业企业64家，规模工业增加值184亿元、增长9%。发放“4321”政银担合作贷款20亿元、担保费率降至1.2%以下，新型农业经营主体“劝耕贷”贷款1.35亿元、试点经验在全省推广，“税融通”贷款6亿元，续贷过桥资金为160家企业提供周转贷款11亿元，中安招商股权投资基金注册落户，与省高新投合作在全省首家设立的中安辰星创业投资基金已定投3个项目3800万元，艾可蓝IPO即将报会，安芯电子已辅导备案，新增“新三板”挂牌企业3家，全年社会融资总量130亿元、增长15%。涉企收费项目减少到82项，社保费率降低3.12个百分点，结构性减税6.6亿元，“营改增”改革为企业减负5.9亿元，17家直供电企业减少用电成本2.6亿元，21家困难企业完成招商重组。在全省率先实现农民进城落户“零门槛”，棚改货币化安置比例88.4%，商品住房去化周期同比缩短6个月。

二是加快“调转促”步伐，产业结构优化升级取得积极进展。组建电子信息、全域旅游、大健康产业三个专家咨询委员会。半导体产业基地列入省第二批战略性新兴产业集聚发展基地，市开发区被评为省优秀电子信息产业园，新增省级“专精特新”中小企业18家、企业技术研发机构10家，新设立博士后工作站1个，新认定高新技术企业26家，新引进3个高层次人才团队在池创新创业，新增国家“万人计划”1人，发明专利授权量200件、增长57.5%，战略性新兴产业产值102亿元、增长17%，高新技术产业产值338亿元、增长15%。整市进入国家首批全域旅游示范区创建名录，编制全域旅游规划及三年行动计划，新增1家国家水利风景区，4家景区通过省4A级旅游景区景观价值评定，九华山机场新增3条航线，联通西安、杭州、昆明、济南、大连、南宁6个城市，池州港新旅游码头建成使用，完成高速公路、国省道及主城区通往4A级以上景区道路沿线旅游标识牌建设，新建、改建旅游厕所50个，开展旅游整体营销81场次，全市接待游客量5100万人次、旅游总收入535亿元，分别增长10%、12%。评选建设第一批最佳摄影点48个，青阳县陵阳镇荣获全国第二家“摄影小镇”。编制大健康产业发展规划，列入国家首批医养结合试点示范单位，成功举办第六届全国绿运大赛、首届健康中国（池州）论坛、首届杏花村文化旅游节、第二届九华山佛文化用品博览会、全国林产品绿色发展论坛，九华山健身瑜伽营地建成开放，九华生命科学研究院落户筹建。建成九华黄精、西山焦枣等6个国家森林生态产品生产基地，新增国家生态原产地保护产品2个、“三品一标”认证10个，中国生态林产品电商交易平台在池州正式上线运营，东至县获批全国电子商务进农村综合示范县。新增省级家庭农场12个、合作社示范社5个，培育现代农业产业化联合体15个。粮食总产67.6万吨。

三是加大美丽池州建设力度，生态宜居特色进一步彰显。编制实施海绵城市建设规划，竣工项目41个，完成投资18.1亿元，建成区域面积9.75平方公里，经受了汛期历史罕见的连续强降雨考验，发挥了明显的示范效应。49项市政重点工程完成投资6.5亿元，城市湿地森林公园养殖户搬迁、绿化、黑臭水体治理和主干道路改造进展加快，建成主城区备用水源工程，改造提升秋浦路、九华山大道等18条城市道路，安装道路隔离栏1.55万米，新建主城区停车场2个，增划停车泊位9500个，新建、改建城镇园林绿地171万平方米、污水管网70公里、燃气管线55公里、道路路灯1260杆，启动实施6个老旧小区整治和100个小区文明创建专项工程，文明城市创建常态化机制基本建立。县城总体规划修编全面完成，“三治三增三提升”行动扎实推进。美丽乡村建设投入资金1.26亿元，改造农村危房4130户，第二批34个省级中心村通过验收，第三批38个、第四批25个省级中心村建设基本完成，创建国家传统村落2个、省级特色小镇1个。望东长江公路大桥建成通车，池州长江公路大桥完成水下基础工程、接线工程全面施工，3条国省干线一级公路和齐石线大中修工程进入路面施工阶段，农村道路畅通工程建成1384公里，青通河航道整治工程全面启动。秋浦河防洪治理工程开工建设，九华河等6处中小河流治理工程、覆盖18万人的农村饮水安全提质增效工程、35座小型水库除险加固主体工程全部完工，长江崩岸治理8.5公里，灾后修复水毁水利工程3647处，全年完成水利投资5.7亿元。首座500千伏涓桥变电站建成投运。全面开展“9+1”城乡环境综合整治和铁腕治矿、重拳治砂、从严治岸、科技治超、精准治污“五治”行动，整顿关闭非煤矿山11家，编制长江岸线保护和利用规划，整合小散码头7座，国省干道超限超载率控制在1%以下，完成总量减排项目41个，淘汰黄标车1973辆，大气环境质量稳居全省前列。三县创建国家生态县通过验收，青阳县列入国家重点生态功能区、获批全国森林旅游示范县，石台县秋浦河源列入国家湿地公园名录。完成全市土地节约集约利用评价，东至县荣获全国国土资源节约集约模范县。

四是全面深化改革扩大开放，发展动力活力日益增强。在全省率先开展整市推进公共服务和行政权力中介服务清单建设试点，市县乡村四级政务服务体系全面建立，企业设立、建设项目办理时间分别压缩至3个、65个工作日。全面实施企业“五证合一”、个体户“两证整合”，工商登记前置审批事项改为后置登记194项，净增私营企业2478户、个体工商户7102户，分别增长18.99%、10.66%。完成发展改革和物价、商务和粮食职能整合，15家市属从事生产经营活动事业单位转企改制稳步推进。农村集体土地确权登记颁证工作全面完成，国有林场改革在全省率先完成，深化供销合作社综合改革全面启动，不动产统一登记制度实现县域全覆盖。

行政机关和参公管理事业单位公务用车制度改革全面完成，城市公交重组工作有序推进。制定出台国有企业改革方案，强化国有资产资金管理，清理盘活财政存量资金4.1亿元，争取地方政府置换债券60.02亿元。推进PPP模式扩围增效，储备项目96个、总投资973.8亿元，4个项目列入财政部第三批PPP示范项目。招商引进规模项目510个、建成投产132个，其中省外亿元以上项目140个、到位资金354亿元，实际利用外资3.65亿美元。江南产业集中区总体规划获批，省级以上园区引进5亿元以上项目8个。民间投资增长6%，民营经济增加值占全市经济比重达64%。全年进出口总值6亿美元、增长15.6%。

五是高度重视保障改善民生，社会和谐稳定局面持续巩固。民生类支出129亿元，占财政总支出的86.4%，33项民生工程和10件惠民实事完成投入31.17亿元。实施脱贫攻坚“十大工程”，制定光伏扶贫、金融扶贫、健康脱贫等政策措施，落实579家单位、近万名干部结对帮扶，投入扶贫资金3.2亿元，发放扶贫贷款918万元，完成649人易地扶贫搬迁，建设63个贫困村、300个贫困户光伏电站，扶持23个乡村旅游扶贫重点村，提前实现农村低保标准和扶贫标准“两线合一”，1.6万贫困人口脱贫，18个贫困村出列。510户倒房群众全部入迁新居。创业就业技能培训11242人，支持初创群体创业1675人，城镇新增就业19548人，农村转移劳动力18556人，城镇登记失业率3.01%。企业退休人员养老金人均月增142元，五保供养标准人均年增500元，城乡低保标准分别提高5%、10%，清退不符合标准的低保对象1191人。保障性安居工程开工2454套、基本建成6421套，山区库区农房保险参保35.47万户。新建、改扩建幼儿园9所，完成义务教育阶段薄弱学校办学条件改善项目60个，“工学一体”招生255人，校外学生接送点规范整治常态化机制基本建立。依托市人民医院组建城市医联体，开展贵池区医共体试点，基本公共卫生服务人均补助标准提高到45元。全面两孩政策稳步有序实施，人口自然增长率6.48‰。《池州市志》正式出版。九华山佛学院恢复办学，江心回民村入选第二批中国少数民族特色村寨。10人当选年度 “中国好人”、“安徽好人”。妇女儿童、老龄、残疾人、红十字等事业取得新成绩，地震、对台、外事侨务、档案、保密、新闻出版、社会科学等工作取得新进展。完成10个城市社区“三社联动”、70个农村社区建设试点。启动“七五”普法，办理法律援助案件2776件。矛盾纠纷排查和信访积案化解力度不断加大，社会治安防控体系进一步健全。安全生产形势持续稳定，全年未发生较大及以上事故。应急管理进一步加强，食品药品安全、物价、市场秩序、网络安全监管有力有序，气象防灾减灾中心基本建成。烈士纪念馆、国防教育展馆建成开放，市光荣院揭牌启用，实现全国双拥模范城“四连冠”。

特别指出的是，面对去年（2016年）“雨情超历史、汛情98+”特大洪涝灾害，我们发扬“心系群众、忘我拼搏、万众一心、善作善成”的抗洪精神，夺取了“长江大堤安然无恙、中小水库安全度汛、地质灾害无人伤亡、转移人员妥善安置、灾害损失降到最低、灾后恢复有力有序”的重大胜利，驻池部队和民兵预备役人员、武警、消防、边防、公安、城管以及全市广大干部群众、社会各界人士为抗洪救灾作出了重要贡献！

一年来，我们扎实开展“两学一做”学习教育，讲看齐、见行动，深入践行“三严三实”，全面加强政府自身建设。政府党组带头认真履行党组工作职责，坚持党组学习会议、民主集中制、党内政治生活等规章制度，严守政治纪律和政治规矩。制定重大行政决策程序规定及8个配套制度，提请市人大常委会审议首部实体性地方性法规《池州市城市管理条例》，制定首部实体性政府规章《安徽升金湖国家级自然保护区管理办法》。自觉接受人大监督，依法执行人大决议决定，办理人大议案、代表建议117件。主动接受政协民主监督，办理政协委员提案302件。全面落实新闻发布会、政策解读制度，完成市政府网站群建设，上线运行政务微信，回复办理市长信箱、市长热线和网民留言6729条。坚持用制度管钱管事管人管资源，健全市直行政事业单位国有资产、办公用房、财政资金、财政预算、编外聘用人员、内部审计等制度体系，加大审计监督和问题整改力度，开展“酒桌办公”等不正之风专项整治。严格落实政府系统党风廉政建设主体责任，加强重大决策和重要工作落实问责追究，抓落实的制度链条进一步健全，政府执行力和公信力进一步提升。

各位代表！过去的一年是极不平凡的一年，成绩来之不易。这是省委、省政府和市委科学决策、坚强领导的结果，是市人大及其常委会和市政协强化监督、社会各界大力支持的结果，是全市干部群众同心同力、克难攻坚的结果。在此，我代表市人民政府，向广大工人、农民、知识分子、干部、离退休老同志，向驻池解放军指战员、武警官兵和政法公安干警，向各民主党派、各人民团体和各界人士，向关心、支持池州发展的中央和省驻池单位，向在我市创业的投资者、建设者，向给予市政府高度信任和倾力支持的各位人大代表、政协委员，表示衷心的感谢！

回顾过去的一年，我们清醒地认识到，发展中仍存在诸多困难和挑战。一是受宏观环境等多重因素影响，实体经济经营困难，产业结构仍然偏重，新经济、新业态发展刚刚破题，支撑效应仍不明显，短期内经济增长趋缓，少数指标与计划目标尚有差距。二是城乡公共基础设施建设欠账较多，群众对医疗、养老、教育、社会保障、城市管理、人居环境等方面还有很多不满意的地方，脱贫攻坚啃硬骨头的难度越来越大。三是政府职能转变仍然艰难，“放管服”改革还有很多不到位、不配套、不衔接的问题，少数干部责任意识、担当意识、服务意识不强，发展环境有待进一步优化，改进作风、提高执行力需要常抓不懈。我们要直面这些问题，认真加以解决，不负人民重托。

二、2017年重点工作

今年（2017年）是党的十九大召开之年，也是实施“十三五”规划的重要一年，是供给侧结构性改革的深化之年。纵观国际国内宏观形势，综合分析自身潜力优势，总体上机遇大于挑战，池州仍处于大有可为的重要战略机遇期。当前，经济发展新常态的特征更加明显，全球经济持续深度调整，新技术革命加速孕育，我市适应新常态、推动新发展迎来重要窗口期。立足新起点、面向新征程，市第四次党代会全面贯彻新发展理念，作出了建设绿水青山和金山银山有机统一排头兵的战略部署，坚持特色发展、错位发展、高端发展，主抓“三大重点产业”，与国家和省实施“中国制造2025”、创新型“三个强省”、全域旅游、健康中国、健康安徽等重大战略方向高度契合，与池州自身区位条件、产业基础、创新资源、生态环境等综合竞争优势充分适应，与新时期信息消费、文化旅游、体育健身、健康养老等新的消费热点紧密相关，蕴含着巨大的增长空间。只要我们坚定地践行新发展理念，扬长补短，持续用力，就一定能够闯出“五大发展”的池州新路，实现“五先”发展的奋斗目标。

今年（2017年）政府工作的总体要求是：全面贯彻党的十八大和十八届三中、四中、五中、六中全会精神，深入贯彻习近平总书记系列重要讲话特别是视察安徽重要讲话精神，坚持稳中求进工作总基调，坚持发展为上、民生为本、脱贫为先、平安为基，围绕“五先”发展目标，抢抓“三区一带一群”建设机遇，扎实开展“五大发展行动”，深入推进供给侧结构性改革，着力构建绿色产业体系，着力补齐城乡发展短板，着力深化改革扩大开放，着力加强保障改善民生，保持经济持续健康发展和社会和谐稳定。

综合考虑，今年（2017年）经济社会发展的主要目标是：全市生产总值增长8%以上，财政收入增长6.5%以上，规模工业增加值增长9%以上，固定资产投资增长12%，社会消费品零售总额增长10%，进出口总额增长5%，城镇常住居民人均可支配收入增长与经济增长同步，农村常住居民人均可支配收入增长高于经济增长，居民消费价格涨幅控制在3%左右，城镇新增就业1.37万人、登记失业率控制在4.5%以内，节能减排达到省控目标。

经济增长预期目标8%以上，体现了“稳”的要求，稳是主基调，是大局、是首要；争取了“进”的突破，稳不是慢作为、不作为，而是要在稳的前提下在主攻方向上有所斩获，在把握好度的基础上奋发进取。工作中，必须着力把握好四点：

一是必须坚持以新发展理念统领发展全局。新发展理念指明了我国经济发展方向，明确了今后经济社会发展的思路和着力点，是我们适应新常态、融入长三角、争当排头兵的战略指引、重要遵循、治本之策。必须更加坚定自觉地用新发展理念统领发展全局，以“五大发展行动计划”为总抓手，一体谋划、一体部署、一体推进，贯穿于经济社会发展全过程和各领域，切实把发展的着力点转到崇尚创新、注重协调、倡导绿色、厚植开放、推进共享上来，围绕行动计划，谋划实施一批重大工程，确保今年（2017年）扎扎实实开局。

二是必须坚持全面融入长三角提升标杆。池州正式列入《长江三角洲城市群发展规划》，标志着我市在国家战略层面全面融入长三角一体化发展。但“纳入”不等于“融入”，“入列”不等于“并列”，我市与苏浙沪在经济基础、体制机制、一体化和国际化水平上都还存在较大落差。必须切实增强加快追赶的紧迫感、责任感和使命感，在全面对标长三角中提升标杆、明确定位，加快产业发展、基础设施、对外开放以及思想观念、精神状态、体制机制方面的等高对接，不断缩小发展差距，奋力在全面融入长三角中干在实处、闯出新路。

三是必须坚持做优产业促进经济提质增效。新理念引领新发展，新常态必须新作为。面对决定经济增长的供需条件、资源环境发生的深刻变化，走老路既行不通又走不远，必须把创新驱动放在发展全局的核心位置，抢抓安徽建设创新型“三个强省”的机遇，坚持生态优先、彰显特色的发展思路，加快发展高附加值的制造业、高带动力的服务业、高品质的特色农业，以增量控制带动存量调整，以动力转换带动结构优化，推动新技术、新产业、新业态加快成长，带动城乡人民实现更加充分、更高质量的就业创业，实现经济效益、社会效益与生态效益的有机统一。

四是必须坚持改善民生增进人民群众福祉。“民之所呼，政之所向”。必须牢固树立以人民为中心的发展思想，按照人人参与、人人尽力、人人享有的要求，坚持问题导向，把工作重心放到加快产业转型升级、优化创新创业环境、加强城市规划建设管理、强化生态建设治理、补齐民生保障短板上来，集中力量做好普惠性、基础性、兜底性民生建设，使全市人民在共建共享发展中增进获得感、提升幸福度，加快建设让全市人民更有认同感、归属感、自豪感的幸福和谐美好家园。

重点抓好以下七个方面工作：

（一）着力抓好供给侧结构性改革，促进实体经济稳定增长

持续扩大有效投入。落实“四督四保”制度，强化“三项清单”管理，实施长九（神山）灰岩矿综合开发、艾可蓝机动车尾气净化产品产业化及工程技术研究中心等省亿元以上重点项目360个，新开工100个，竣工65个，完成投资260亿元以上。组织实施市本级政府性投资项目88个，完成投资30.57亿元。落实扩大民间投资政策措施，继续推出一批政府与社会资本合作项目，释放民间投资潜力。强化园区招商、产业链招商、精准招商、以商引商，完善招商引资绩效考核办法，建立重点招商项目全程代办制，力争在牵动性龙头项目招商上取得突破，全年引进项目560个，其中省外亿元以上项目135个、到位资金390亿元，利用外商直接投资3.9亿美元，签约项目转化率60%以上。制定工业园区转型升级考核评价办法，全市园区工业总产值增长12%、税收增长8%以上。围绕补齐产业发展、基础设施、公共服务、生态环境等短板，加强重大项目谋划，确保列入省重点建设项目、中央预算内投资项目不低于应有份额。

深入实施创新驱动发展战略。健全技术和产业、资本和金融、平台和企业、制度和政策等创新支撑体系，实施10项市科技重大专项，力争引进和扶持3个高层次领军人才团队在池创新创业，组建1个主导产业技术创新战略联盟，新认定高新技术企业15家、省级企业研发平台10家、省级科技孵化器（众创空间）2家，发明专利授权量增长10%以上，高新技术产业增加值增速超过全省平均水平。支持池州高新区申报创建国家级高新区。推进传统产业新兴化发展，实施100项企业技改和3家企业“机器换人”、智能工厂改造项目，完成技改投资230亿元，新增规模工业企业50家、省“专精特新”中小企业10家。创新国际市场开拓方式，推进出口品牌建设，健全外贸企业孵化培育机制，建设外贸综合服务平台，推进外贸“优进优出”，新增外贸实绩企业15家。

进一步推进“去降补”。坚持和完善精准帮扶重点企业制度，支持15家以上困难企业完成招商重组。全面落实降低实体经济企业成本各项措施，积极争取扩大直供电交易覆盖范围，完善续贷过桥、政策性融资担保风险补偿机制，新增“4321”新型政银担合作贷款20亿元、税融通贷款6亿元以上，“劝耕贷”实现乡镇全覆盖。鼓励支持新市民住房消费和农民进城购房落户，确保主城区棚改货币化安置100%、各县不低于80%，商品房去化周期控制在14个月之内。

（二）着力加快特色主导产业发展，培育经济发展新动能

加快战略性新兴产业集聚发展。推进战略性新兴产业“一号工程”省级半导体产业基地建设，创建先进半导体研究院等3家省级以上创新平台，开工建设年产1.5万吨电子铜箔三期、高端封测产业园、氮化镓半导体材料研发及产业化等一批龙头项目，建成安芯电子二期6-8英寸晶圆制造、超元半导体项目二期、钜芯半导体晶圆制造和封测项目二期、赛米科封装测试等一批重点项目，确保投资、产值、税收等关键指标达到省考核要求。启动2-3个市级产业集聚发展基地建设，力争氮化镓单晶衬底、碳纤维材料等2-3个项目列入省重大新兴产业专项、重大新兴产业工程。战略性新兴产业产值增长15%以上。

加快旅游全域化进程。全面实施全域旅游发展规划，启动九华山风景名胜区新一轮总体规划编制，完成平天湖风景区总体规划修编，依据规划市县区分别确定1个“头号项目”强力攻坚，力争取得实质性突破，带动规划项目整体实施。抢抓国家支持长江黄金旅游带、皖南国际文化旅游示范区实施旅游休闲重大工程机遇，发挥好政府资金“四两拨千斤”的引导作用，建设主城区和各县旅游集散中心，消除重点景区“断头路”，启动“一主四环”最美自驾公路、自驾车主题营地及服务驿站、直升机场规划建设，在A级旅游景区、重点乡村旅游区、旅游扶贫重点村等地新建和改扩建一批旅游厕所、停车场、交通引导标识，建成全市智慧旅游综合云平台。实施旅游综合执法体制改革，加快形成旅游市场综合监管格局。组建全市旅游营销联盟，做大皖南国际文化旅游示范区展销中心，继续办好佛文化用品博览会、杏花村文化旅游节、全国摄影大展等展会节庆活动，积极融入“一带一路”国际旅游合作。旅游接待游客、收入分别增长10%、12%以上，服务业增加值增长9%以上。

大力培育大健康产业。全面实施大健康产业发展规划，突出补齐产业发展短板，制定产业促进政策，推进国家医养结合试点，规划建设1-2个特色大宗中药材种植基地，实施2个以上医疗养老联合体项目，大力发展以中医药健康养老为特色的护理院、疗养院等专业医疗机构，积极引进建设全科医生培训基地、高等医学院校分院分校。统筹规划，合理布局，推进各县区因地制宜，特色错位融合发展，重点加快九华山健康文化园、江南产业集中区健康制造、贵池绿色中药材、东至健康食品、石台富硒功能农业、青阳九华黄精等健康产业基地规划设计和项目集聚，启动杏花村绿运休闲小镇、九华山禅修小镇等健康小镇规划建设，力争每个县区引进建设1个5亿元以上标志性项目。积极扩大健康品牌赛事展会影响，力争全国绿运大赛赛事升格，继续办好环平天湖国际马拉松大赛、健康中国（池州）论坛，推进升金湖环湖景观公路（自行车赛道）规划建设。全面开展硒资源普查，编制实施富硒产业专项规划。

推进现代生态农业产业化。加快建设现代农业产业体系，优化农产品结构，推进农村一二三产业融合发展，新增“三品一标”认证10个，引进5个5000万元以上农产品加工项目，实现规模以上农产品加工产值125亿元；实施休闲农业与乡村旅游提升工程，创建省级示范点1个，实现休闲农业营业收入28亿元；积极创建省级以上蔬菜标准园，加快“菜篮子”流通体系建设，切实降低市场菜价。加快建设现代农业生产体系，实行最严格的耕地保护和基本农田保护制度，建设高标准农田11.36万亩，稳定粮食生产；加快构建公益性和经营性相结合的农业社会化服务体系，推动龙头企业开展物联网技术示范应用，主要农作物耕种收机械化率比上年提高1个百分点；扩大农业保险覆盖面，加强重大动植物疫病防控，开展农产品质量安全可追溯试点。加快建设现代农业经营体系，新增省级示范家庭农场10个、合作社示范社5个，培育现代农业产业化联合体10个；推进东至县、石台县国家电子商务进农村示范工程建设，加快县级电子商务综合服务体系全覆盖，力争中国生态林产品电商交易平台线上交易额突破10亿元。

（三）着力补齐基础设施短板，提升城市和产业承载功能

加快完善外联内通综合交通体系。编制实施综合交通发展规划，完成池州长江公路大桥下部构造和接线路基工程，建成东流至尧渡、木镇至青阳、高岭至牛头山一级公路，加快青通河航道整治工程疏浚施工，配合实施大渡口至江口段长江干流航道整治，开工建设池州至殷汇一级公路改建、南陵界至木镇一级公路，加快铜九铁路电气化改造、宿松至宣城城际铁路池州段、石台至查桥、青阳至桃花潭公路、秋浦河航道整治等项目前期工作，力争池州长江公铁大桥可研报告报批。组建两个专班，推进武杭高铁池州至黄山段、济祁高速殷汇至石台段前期工作，力争年内开工。提升池州港港口功能，推动牛头山港区水运口岸对外开放，加快发展区域性物流中心。加快九华山机场扩建改造，力争开通境外直航包机。

加强城市规划建设。按照彰显特色、多规合一的要求，启动市、县空间规划编制，开展主城区控制性详细规划动态调整，完成地下综合管廊规划、公共服务设施综合规划、城市空间特色规划等专项规划编制，严格按照规划组织实施，切实维护规划的严肃性和权威性。编制城市湿地森林公园详规，启动绿运主题公园建设。加快清溪河流域水环境综合整治、滨江区及天堂湖新区棚改基础设施PPP项目建设，基本消除主城区10处黑臭水体，实施城市道路“白改黑”40公里，新建、改建燃气管线50公里、供排水管道45公里，完成国家海绵城市建设试点任务。启动城市排水防涝三年行动，全面开展城市易涝点综合整治，力争到2019年汛前主城区和各县城基本消除城市内涝隐患。开展城乡建设绿色发展六项重点行动，推进绿色生态城市综合试点，新增和改造园林绿地面积70万平方米，30%以上城镇新建建筑达到绿色建筑标准，力争青阳县建成省级园林县城。加快省级以上开发区按城市基本公共服务标准完善配套功能。

提升城市管理水平。贯彻执行《池州市城市管理条例》，深化城市执法体制改革，完成市、县政府城市管理领域的机构综合设置，实施主城区环卫、市政公用设施管理体制改革，建成社会服务管理信息化平台，全面运行数字化城市管理平台，完成城市快速路和主干道智慧交通建设，开展“两治三改”三年专项行动，加大市容市貌、交通秩序、物业管理整治力度，依法严厉打击城市违法建设行为。创建省级卫生城市。建立健全文明城市创建“八项工作机制”，深入实施“七大提升工程”，力争迈入全国文明城市行列，实现省级文明县城全覆盖。

深入推进美丽乡村建设。完成县域村庄规划编制，加强村镇规划建设管理队伍建设。启动第五批54个省级中心村建设，实施16个乡镇政府驻地建成区环境整治，实现农村生活垃圾治理全覆盖，镇污水集中处理率达到30%。启动省级特色小镇培育，加强传统村落保护。实施灾后水利水毁修复与薄弱环节建设性治理三年行动方案，完成秋浦河防洪治理主体工程、尧渡排涝泵站土建工程，开工建设20座小型病险水库除险加固工程、5处中小河流治理工程，加快东湖圩、唐田圩、童埠圩等重点险工险段除险加固达标，确保安全度汛。继续实施农村小型水利工程改造提升行动计划。深化农村公路管理体制改革，推进“四好农村路”建设，建成农村道路畅通工程598公里。推进220千伏东至汇流站等8个骨干电网和农村电网改造升级工程建设。

（四）着力加强生态治理保护，增创美丽池州新优势

强力推进“五治”行动。高标准贯彻大气、水、土壤污染防治行动计划，启动全市统一的生态环境监测数据平台建设，建立精准治污清单。常态化开展“9+1”城乡环境综合整治，加强主城区烟花爆竹燃放源头管理，全面淘汰黄标车，秸秆综合利用率达到89%，大气环境质量完成省定目标。全面推行“河长制”，实行最严格的水资源管理制度，强化饮用水源保护，加强主城区二次供水水质监测，全面实施城市禁养区内畜禽禁养，申报升金湖国家重要湿地保护工程项目，完成平天湖水环境整治项目，加快清溪、城东污水处理厂提标改造，水功能区水质达标率93%以上。推进林业增绿增效行动，新造林3.67万亩，治理水土流失20平方公里。加大农业面源污染防治，扎实推进不使用化学农药和化肥乡镇试点并逐步扩大范围，抓好土壤污染防治和固体废物、城市生活垃圾无害化处置，推进畜禽养殖废弃物综合利用和无害化处置。开展矿山综合整治和绿色矿山创建攻坚，整顿关闭非煤矿山8家，创建绿色矿山31家。深入开展长江、内河河道采砂“两禁一清”专项整治，确保秋浦河、九华河、青通河等干流河道全面禁采。全线开展砂场清理、小散码头整合和岸线功能区整治，促进岸线资源规范管理和有序开发。加大科技治超力度，实现省、市、县三级源头治超联网联控。

加快低碳循环发展。全面落实主体功能区规划，划定生态保护红线。推进国家生态文明建设示范市、全国绿化模范城市和市开发区国家低碳工业园区创建，完成国家低碳城市试点任务，力争平天湖国家湿地公园通过验收。加大闲置土地处置力度，确保前5年新增建设用地供地率70%以上。严格环境准入和污染物总量排放控制，加强钢铁、水泥、火电、污水处理厂等重点行业企业减排项目和达标排放监督管理，开展工业领域能效提升行动，实施园区集中供热、贵航特钢二期余热发电、东至经开区热电联产等一批节能项目，加快新能源汽车充电桩布局建设，积极发展风电、光伏发电等清洁能源。

（五）着力深化重点领域改革，激发市场活力和社会创造力

深化“放管服”改革。全面实施“3+2”清单制度，建成运行政府权力运行监管平台。转变政府投资管理职能，上线运行政务服务网上大厅，落实企业投资项目通过在线监管平台办理制度。全面实现行业协会商会与行政机关脱钩，大力引进和培育中介机构。建成市县一体化公共资源电子交易平台，实现全程电子化、服务标准化、监管智能化，严厉打击挂靠借用资质等招投标违法违规行为，健全“黑名单”制度。启动中小企业信用体系建设，开展贵池、东至、石台农村信用体系建设试点。深化商事制度改革，加强事中事后监管，全面推开“双随机、一公开”模式。建立编制周转池管理制度，完善高层次人才柔性引进机制，深化“创业池州”行动。

深化财政金融体制改革。加强全口径预算管理，全面开展预算评审论证工作，推进预算信息公开，提高预算编制绩效。加强全口径政府债务管理，清理盘活财政存量资金，积极争取地方政府置换债券，组建资产管理公司，有效防范金融风险。加强财政收支管理，完善财税征管分析和协税护税机制，强化县区预算监督，确保财政收支平衡，将有限的资金用在“刀刃”上。推进企业上市挂牌，力争1家企业IPO上市、1家企业报会在审、1家企业上市辅导、3家企业“新三板”挂牌，直接融资30亿元以上，社会融资总量140亿元。发挥政府引导基金作用，引进设立PPP引导基金、绿色发展产业基金、健康养老服务产业投资基金，积极引导各类基金开展股权投资。

深化国资国企改革。分类推进国有企业改革，整合国有股权，组建基础建设投资集团、资产运营集团、旅游投资集团和杰达资产经营集团四大国有资本（资产）公司。完成国有企业负责人薪酬制度改革，启动事业单位和国有企业公务用车制度改革。推进国资监管机构职能转变，优化国有资本监管方式，深化国有企业和国有资本审计监督，完成15家市属从事生产经营活动事业单位转企改制，实现国有资产出资人监管全覆盖。

深化农村综合改革。实施农村土地所有权、承包权、经营权分置改革，巩固农村土地确权登记颁证成果，全市适度规模经营面积比重达到45%。稳妥推进承包土地经营权和农民住房财产权抵押贷款、农村宅基地制度等改革试点，完成4个村的农村集体资产股份合作制改革试点，选择1个村开展“资源变资产、资金变股金、农民变股东”改革试点。全面推开农业“三项补贴”改革。

（六）着力保障改善民生，增进人民群众福祉

全力打好脱贫攻坚战。按照脱贫攻坚“四个全覆盖”要求，严格落实扶贫开发工作责任制和包保帮扶制度，统筹推进脱贫攻坚“十大工程”，建设11个贫困村、1000个贫困户光伏电站，完成年度易地扶贫搬迁任务，积极开发扶贫辅助性岗位，贫困人口基本实现医疗兜底保障，确保22832人脱贫、24个贫困村出列。建立健全返贫预警制度，强化返贫信息管理，及时将返贫户纳入帮扶范围，对已脱贫的村、户一定时期内继续保留原有扶贫政策，对未考上高中或大学的贫困家庭子女实现免费中等职业教育和“雨露计划”全覆盖。

持续加强民生保障。继续实施33项省民生工程，加强民生资金绩效评价，健全常态长效管养机制。深入推进“工学一体”就业就学模式，开展创业就业和新型职业农民培训1.25万人，农村劳动力转移就业1.8万人。推进机关事业单位养老保险制度改革。完成全民参保登记计划，完善社会保险转移接续政策，加快城乡基本医疗保险制度整合，推进基本医疗保险异地就医实时结算，开展特殊困难群体和重点优抚对象等老年人意外伤害保险统保工作。加强城乡低保信息化动态管理，提高农村五保、孤儿、优抚对象保障水平，全面落实临时救助制度。保障性安居工程开工1022套、竣工1323套，分配入住12536套。选择10个以上公办养老机构推行公建民营试点，市老年公寓投入使用。

协调发展社会事业。启动第三期学前教育行动计划，力争全市90%以上幼儿就读普惠园。谋划实施主城区基础教育三年提升行动，完成78所农村义务教育阶段薄弱学校办学条件改善项目。推进考试招生制度改革。加快发展现代职业教育，推进高等院校和职业学校政校企联动、产学研融合，提升地方院校服务地方经济发展的功能，支持安徽人口职业学院二期扩规。规范发展民办教育，扶持特殊教育，支持老年教育。深化医药卫生体制综合改革，实施临床路径管理，推进按病种付费，启动青阳县医共体试点，推广家庭医生签约服务，完善分级诊疗体系。加强社区卫生服务中心和乡镇卫生院能力建设，确保市儿童医院、妇幼保健院主体工程完工。加强重大传染病、职业病防控。扎实做好人口和计生工作，保持适度生育水平。力争市图书馆建成国家一级馆。编制池州古道保护和利用规划，实施榉根关等古道保护项目，加强文物保护，常态化举办非物质文化遗产展演。做好新经济统计调查工作，抓好第三次全国农业普查，推进依法统计、依法治统。加强外事侨务、对台、保密、档案、地方志、广播电视、新闻出版、社科研究等各项工作。

（七）着力提升社会治理水平，维护社会和谐稳定

夯实社会治理基础。完成10个城市社区“三社联动”试点，巩固农村社区建设试点成果，依法做好第十届村委会换届选举工作。深入推进“七五”普法，提高“一村一法律顾问”覆盖面。推进信访法治化，及时解决群众合理诉求。大力培育和践行社会主义核心价值观，持续提升市民文明素质。充分发挥工会、共青团、妇联等人民团体的重要作用，支持关心下一代工作和红十字、慈善公益事业，切实维护妇女、儿童和老年人、残疾人合法权益，启动市老年活动中心、妇女儿童活动中心、青少年活动中心建设前期工作。

强化公共安全管理。完善社会治安综合防控体系，完成公安视频监控“天网工程”二期建设，深化“守护平安”专项行动，严厉打击违法犯罪活动。严格落实安全生产责任制，深入开展“铸安”行动，抓好危险化学品重点县安全生产攻坚工作，坚决遏制重特大安全事故。强化食品药品安全、产品质量、市场物价、建设工程质量监管。完善监测预警应急机制，建成防灾减灾、应急管理等统一指挥系统。依法维护民族团结和宗教和谐稳定。支持驻池部队建设，加强国家安全、国防教育、国防动员、民兵预备役和人民防空工作，推进军民深度融合发展，争创全省双拥模范城“五连冠”。

三、进一步加强政府自身建设

面对新形势、新任务，必须以新理念统领政府工作、以高标准加强自身建设，不忘为民初心，勇担历史使命，努力建设人民满意政府。

（一）增强干事创业思想自觉。牢固树立“四个意识”特别是核心意识、看齐意识，坚定理想信念，增强“四个自信”，用理想之光、信仰之力，全面激发各级干部干事创业的激情和斗志。始终紧扣发展这个第一要务，牢记为民宗旨和组织重托，以先发地区树立和践行新发展理念的先进经验为参照系，全面审视，自觉对标，坚决破除思想桎梏、小富即安、按部就班、畏首畏尾，以只争朝夕的精神抓好每一天、干好每件事、交好每份答卷。

（二）全面推进法治政府建设。健全行政决策机制，加强专家智库建设，进一步完善决策咨询、风险评估、法律顾问、实施后评价制度。坚持依法行政，规范行政执法行为，加强复议应诉工作。自觉接受人大的法律监督和工作监督，主动接受政协的民主监督和社会各界的监督，配合做好地方立法工作，认真办理人大代表议案、建议和政协委员提案，充分听取和吸纳各民主党派、工商联、无党派人士及各人民团体意见。加强政务公开和政府信息公开，积极引导和妥善应对网络舆情，及时回应社会关切。

（三）完善狠抓落实工作机制。坚持学以致用，善于在中央政策精神中找指针、找方法，转化为池州发展的科学思路、创新举措，掌握推动发展的主动权。健全重点工作、重点项目目标责任、台账管理、动态管控、运行调度、督查考核、追责问责机制，持续深化机关效能建设，坚决治理“庸懒散拖”，促进各级干部增强担当意识、服务意识，提高办事效率、工作质量，确保政府工作掷地有声、落实见效。坚持“三个区分开来”，建立容错纠错机制，正确把握失误与错误、为公与谋私的政策和纪律界限，最大限度保护和调动广大干部干事创业的积极性、主动性、创造性。

（四）切实加强全面从严治政。认真贯彻党内政治生活若干准则和党内监督条例，严格落实中央八项规定精神和省、市相关规定，切实履行政府系统党风廉政建设主体责任，加强行政监察、审计监督，全面推行自然资源资产离任审计试点，加大政府投资建设项目跟踪审计力度，坚决惩治侵害群众利益的不正之风和腐败行为。加强政府班子自身和公务员队伍的教育、管理和监督，切实做到秉公用权、依法用权、为民用权、廉洁用权。

各位代表！融入长三角前景可期、催人奋进，争当排头兵重任在肩、时不我待。让我们紧密团结在以习近平同志为核心的党中央周围，在省委、省政府和市委的坚强领导下，凝神聚力，同心同德，奋力争当绿水青山和金山银山有机统一的排头兵，以优异成绩迎接党的十九大胜利召开！
